# PD-L1 Immunohistochemical Detection in Tumor Cells and Tumor Microenvironment: Main Considerations on the Use of Tissue Micro Arrays

**DOI:** 10.3390/ijms17071046

**Published:** 2016-06-30

**Authors:** Gerardo Botti, Giosuè Scognamiglio, Monica Cantile

**Affiliations:** Pathology Unit, Istituto Nazionale Tumori Fondazione “G. Pascale”, via Mariano Semmola, 80131 Napoli, Italy; g.botti@istitutotumori.na.it (G.B.); giosco80@gmail.com (G.S.)

**Keywords:** PD-L1 immunohistochemical (IHC) detection, tumor microenvironment, TMA

## Abstract

PD-1/PD-L1 (programmed death 1/programmed death ligand 1) pathway plays a critical role in immune escape of tumor cells. Recent studies have described that PD-L1 is heterogeneously expressed in various types of cancer, although its prognostic/predictive value is still uncertain. These problems are partly due to a not well defined operating protocol for its detection by immunohistochemistry, but also because most of the studies conducted on large case series were made by Tissue Micro Array (TMA). We are going to discuss this latter point, to highlight that TMA must be set up in an appropriate manner, especially for some markers, such as PD-L1, which, besides being poorly expressed in tumor cells, can be expressed by cells of the tumor microenvironment.

## 1. To the Editor

PD-L1 is a checkpoint receptor that plays an immune-regulatory role in T-cell activation, tolerance and immune-mediated tissue damage [1]. Several studies have recently shown that the PD-1/PD-L1 pathway may have a key role in the interaction of tumor cells with host immune response, and PD-L1 expression in tumor cells may function as a mechanism of adaptive immune resistance. This mechanism of “immune-escape” allows tumor cells to limit T-cells activity in the tumor microenvironment [2].

Despite the importance of PD1/PD-L1 interaction in tumor elusion, the exact mechanism of how PD1/PD-L1 interaction affects tumor microenvironments to promote the escape of tumor cells from anti-tumor immuno-surveillance is not clear. In addition, many inaccuracies were often provided about the prognostic/predictive role of PD-L1 in cancer.

Tumor expression of PD-L1 was associated with cancer progression and poor prognosis of various human cancers [3], but the data available in literature are not uniform and are often conflicting. This problem might be, in part, associated with the use of different clones of antibodies, with variable specificity, and mainly with a score that is not uniquely defined, but also because most of the studies carried out on large case series have been performed on a Tissue Micro Array (TMA) [4,5,6,7].

In fact, although the application of TMA has completely revolutionized biomedical research for certain proteins, such as membrane receptors, cancer stem cells markers, and others with a low range of expressions, this technology should be used in an appropriate manner.

Usually, PD-L1 is present on tumor cells with a very low expression level, and with a heterogeneous distribution in different tumor types [8]. Often, its expression is more evident on the invasive tumor front, barely represented in TMA cores.

Moreover, PD-L1 could also be expressed in the tumor microenvironment, in particular in infiltrating lymphocytes (TIL), monocytes and macrophages [9]. Taube et al. [9] found that the expression of PD-L1 in tumor cells and immune cells was highly associated with PD-1 expression in infiltrating lymphocytes, and had the strongest association with response to nivolumab. This data suggested to measure, not only PD-L1 tumor cell positivity, but also PD-L1 expression in immune-infiltrating cells, for prediction of immune checkpoint therapy response.

This also represents an important limitation in the use of TMA, where cores are generally chosen within the tumor area and are not sufficiently representative of the tumor microenvironment.

We have recently had the opportunity to analyze the expression of PD-L1 on a large casuistry of different tumor types. In selected cases, we compared the expression of PD-L1 in TMA cores and in single/whole sections of the same samples. This has allowed us to re-evaluate, in most of the cases, the expression, assigning values in terms of positive cells percentage completely different from those assigned to TMA cores.

**Figure 1:**
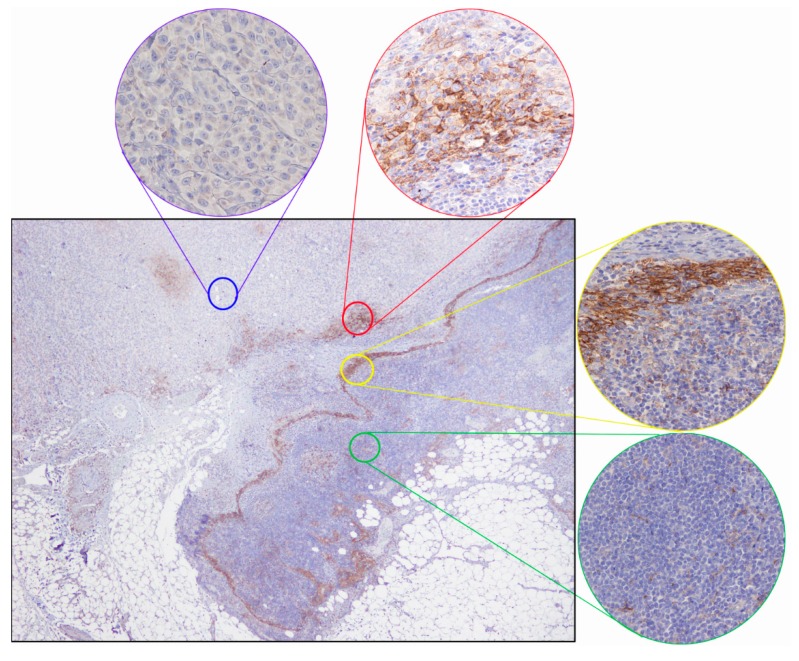
Immunohistochemical staining of PD-L1 in a melanoma sample: Whole section image (20×) with positive tumor cells (detail in red circle, 400×) and positive lymphocytes (detail in the yellow circle, 400×) on invasive tumor front, negative tumor cells (detail in blue circle, 400×), and negative lymphocytes in tumor microenvironment (detail in green circle 400×).

Moreover, in some cases, cores that originally were totally negative for PD-L1 expression showed areas of positivity in the whole sections. In particular, we detected a positive staining of tumor cells (Figure 1, detail in the red circle) and lymphocytes (Figure 1, detail in the yellow circle) on the invasive tumor front, while some of the selected cores used for the assemblage of TMA showed a negative staining for tumor cells (Figure 1, detail in the blue circle) and for lymphocytes in tumor microenvironment (Figure 1, detail in the green circle).

## 2. Conclusions

Our personal experience in TMA employment for PD-L1 detection in some solid human tumors, has allowed us to define the inadequacy of this technique for the its detection, suggesting to analyze the whole section, or to consider, during the preparation of the TMA, the use of multiple cores to be representative of the entire tumor area and the tumor microenvironment [10].

In conclusion, we suggest, in addition to standardization of immunohistochemical (IHC) protocols for the detection of PD-L1 in tumor cells and tumor microenvironments of different cancer subtypes with a definition of adequate cut-offs, to also evaluate its expression in the whole section, to correctly define the real prognostic and predictive value of this marker in tumor disease.
